# Influence of Calcium Silicate‐Based Cement‐Repaired Root Canals on Push‐Out Bond Strength of Glass Fiber Posts: An In Vitro Study

**DOI:** 10.1002/cre2.70340

**Published:** 2026-03-26

**Authors:** Yasudthama Rattana‐aporn, Weeranuch Thong‐ngarm

**Affiliations:** ^1^ Phatthalung Hospital Phatthalung Thailand; ^2^ Division of Crowns and Bridges, Department of Restorative Dentistry and Periodontology, Faculty of Dentistry Chiang Mai University Chiang Mai Thailand

**Keywords:** calcium silicate‐based cement, glass fiber posts, push‐out bond strength

## Abstract

**Objectives:**

This study aimed to compare the push‐out bond strength of glass fiber posts cemented with self‐etch and self‐adhesive resin cements in various sizes of perforated roots repaired by calcium silicate‐based cement.

**Materials and Methods:**

Human premolars were divided into six groups based on perforation size (none, 0.8 mm, or 1.4 mm) and type of resin cement: self‐etch adhesive (SE) and self‐adhesive (SA) (*n* = 12). Root perforations were created using a cylindrical diamond bur and repaired with calcium silicate‐based cement, then stored in distilled water at 37°C for 7 days. Fiber posts were cemented with either SE or SA resin cement. Specimens were embedded in epoxy resin, stored for 24 h at 37°C, then sectioned into 2 ± 0.1 mm slices. Push‐out testing was conducted using a universal testing machine at 1 mm/min. Data were analyzed by two‐way ANOVA and Tukey's test (*p* < 0.05). Failure modes were examined under SEM.

**Results:**

Perforation size significantly influenced bond strength. The 1.4 mm group showed lower bond strength (13.07 ± 3.01 MPa) than the 0.8 mm and control groups (16.74 ± 2.63 MPa and 17.13 ± 2.89 MPa; *p* < 0.05). However, the type of resin cement did not significantly affect bond strength between groups (SE: 15.00 ± 3.63 MPa; SA: 16.23 ± 2.96 MPa; *p* > 0.05).

**Conclusions:**

Larger root perforations repaired with calcium silicate‐based cement result in reduced push‐out bond strength of fiber posts. No significant difference in bond strength was found between SE and SA resin cement.

## 1 Introduction

One complication that affects the prognosis and leads to restorative failure of an endodontically treated tooth is root canal perforation. The causes of perforations can be root caries, root resorption, or iatrogenic error (Saed et al. 2016). Perforation allows bacterial leakage from the open environment of supporting tissue to the closed environment of the root canal. When root perforation occurs, root reparation is needed to improve the prognosis. Mineral trioxide aggregate (MTA), a calcium silicate‐based cement, is commonly used for repairing root perforations. Clinical evidence has demonstrated that using MTA for root repair results in a high rate of treatment success (Siew et al. 2015). However, this material has many limitations, including handling difficulties, an extended setting time, and the potential to cause tooth discoloration. Biodentine has been developed and has some properties that are superior to MTA (Kaur et al. 2017; Estrela et al. 2018; Camilleri et al. 2013; Parirokh and Torabinejad 2010).

For an endodontically treated tooth that requires a post and core with crown restoration, a fiber‐reinforced composite (FRC) post is widely used due to its many advantages, especially esthetics. Furthermore, an FRC post has a modulus of elasticity close to that of dentin and a lower incidence of root fracture than a metal post (Goracci and Ferrari 2011). On the other hand, the study of Hamitoglu and Ozkurt‐Kayahan (2025) have found that fiber posts do not result in a higher incidence of root fractures compared to posts made from materials with a higher elastic modulus than dentin, such as zirconia or titanium posts. However, the most frequent report of FRC post failure is post‐debonding (Goracci and Ferrari 2011; Lamichhane et al. 2014). The bonding quality between FRC post, resin cement, and root canal dentin plays an important role in the success of the restoration, especially when perforated root dentin is replaced by root‐repair material.

Recent studies have shown that bioceramic sealers and repair materials can influence the interfacial adaptation and bond strength of FRC posts, and their behavior may differ from that of conventional epoxy resin–based sealers and repair materials (Nesello et al. 2022; de Morais et al. 2023; Alghazaly et al. 2024). There are few studies on the retention of FRC posts in repaired perforated roots. Most previous studies have used MTA, and only a limited number have evaluated Biodentine, particularly with regard to different perforation sizes (Pereira et al. 2016; Kubo et al. 2013; Tavşan and Simşek 2021). The objective of this study was to evaluate the push‐out bond strength of FRC posts in Biodentine‐repaired roots with different perforation sizes, cemented using two resin‐cement systems.

## 2 Materials and Methods

**Figure 1:**
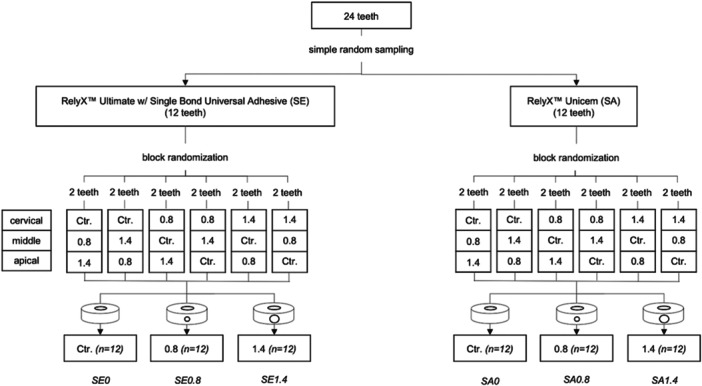
Diagram of the experimental groups in this study.

The present study received ethical approval under No. 22/2019 from the Human Experimentation Committee, Faculty of Dentistry, X University, X (country). Twenty‐four human premolars were selected; they all had one straight root canal, a similar size, a root length of 15 mm, a round canal configuration, and a root canal diameter that was not more than 1.5 mm. All teeth were cleaned using an ultrasonic scaler and then preserved in a 0.1% thymol solution. The teeth were randomly divided into two groups based on the type of resin cement used. The SE group used the self‐etched mode of universal adhesive before being cemented with resin cement (Rely X Ultimate with Single Bond Universal Adhesive, 3M ESPE, Seefeld, Germany). The SA group used self‐adhesive resin cement (Rely X Unicem, 3 M ESPE, Seefeld, Germany). Both resin cement groups were divided with block randomization of each root level to three subgroups (*n* = 12) according to perforation sizes: no perforation (control group), 0.8 mm perforation diameter, and 1.4 mm perforation diameter (Figure 1).

The teeth were decoronated, and root canals were prepared at a length of 10 mm with a peeso reamer (Jota AG, Rüthi, Switzerland) no. 1–3. The specimens were sectioned into three levels, each 2 mm in thickness, starting from the proximal cemento‐enamel junction. The roots were then positioned in the root‐holder apparatus to determine the perforation site at the midpoint of each root level. A cylindrical diamond bur (Jota AG) was used to create a 0.8 or 1.4 mm perforation perpendicular to the root surface. Both perforation sizes were prepared within the same tooth to control for potential variations in mineralization among the teeth included in this study. A cylindrical stainless‐steel rod, wrapped circumferentially with Teflon tape, was inserted into the prepared root canal to achieve maximal adaptation to the canal walls. Biodentine was prepared according to the manufacturer's guidelines and applied to seal the perforations. After allowing it to set for 12 min, the repaired teeth were stored in distilled water at 37°C with 100% humidity for 7 days.

Root canals were finally prepared with a FibreKleer 4x post drill (Pentron, Wallingford, CT, USA) no. 3, 1.5 mm diameter, and 15 mm length. FRC posts (FibreKleer 4x) were cleaned with 70% alcohol, dried with air, and then treated with silane (RelyX Ceramic Primer; 3M ESPE, St. Paul, MN, USA) for 60 s before being air dried again (Muniz and Mathias 2005).

**Figure 2:**
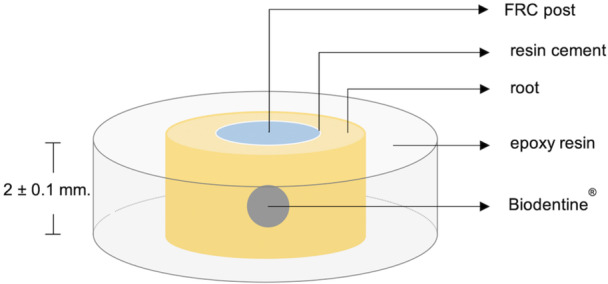
Specimen preparation before the push‐out test.

FRC posts were cemented in root canals with resin cement with the aid of an elongation tip and according to the manufacturer's guidelines. In the SE group, Single Bond Universal Adhesive was applied for 20 s and followed by Rely X Ultimate. For the SA groups, posts were cemented with Rely X Unicem. The post was inserted into the root canal, excess cement was carefully removed, and light curing was performed using a Bluesphase LE curing light (Ivoclar Vivadent, Schaan, Liechtenstein) for 40 s, followed by an additional 6 min for self‐curing. The bonded root specimens were embedded in epoxy resin and kept in distilled water at 37°C with 100% humidity for 24 h. The roots were then sliced into sections of 2 ± 0.1 mm thick using an IsoMet low‐speed precision cutter (Buehler, Lake Bluff, IL, USA) (Figure 2). The push‐out bond strength was tested using a universal testing machine (UTM; Instron 5566; Instron, Norwood, MA, USA), applying force from the apical to coronal direction at a crosshead speed of 1 mm/min. The maximum load of each specimen was recorded in Newtons (N), then converted into megapascals (MPa).

$$\text{Push}-\text{out}\;\text{bond}\;\text{strength}\;\left(\text{MPa}\right)=\frac{\text{Maximum}\;\text{load}\;\left(N\right)}{\text{Post}\;\text{surface}\;\text{area}({\text{mm}}^{2})}$$

The failure modes of the specimens were examined using scanning electron microscopy (SEM) at 50× magnification. Statistical analysis was performed using two‐way ANOVA to evaluate differences between treatment groups. One‐way ANOVA followed by Tukey's multiple comparison test (*p* < 0.05) was used to compare the means among the six groups.

## 3 Results

Mean push‐out bond strengths and standard deviations are shown in Table 1. Statistical analysis indicated that the only factor that affected bond strength was perforation size, and not the type of resin cement (Tables 2 and 3).

**Table 1:** Mean and standard deviations of push‐out bond strength in study groups.

Cement	Perforation diameter (mm)	Push‐out bond strength (MPa)
RelyX Unicem (SA)	0	17.32 ± 3.17^a^
	0.8	17.06 ± 2.68^a^
	1.4	14.51 ± 2.35^ab^
RelyX Ultimate (SE)	0	16.94 ± 2.70^a^
	0.8	16.42 ± 2.65^a^
	1.4	11.64 ± 2.98^b^

*Note:* Different superscript letters indicate a statistical difference.

**Table 2:** Two‐way ANOVA of between‐subject effects.

Dependent variable: Push‐out bond strength					
Source	Type III sum of squares	df	Mean square	*F*	Sig.
Corrected model	292.725^a^	5	58.545	7.650	0.000
Intercept	17624.823	1	17624.823	2303.111	0.000
Cement type	30.189	1	30.189	3.945	0.051
Perforation size	239.981	2	119.990	15.680	0.000
Cement × perforation size	22.555	2	11.278	1.474	0.237
Error	505.073	66	7.653		
Total	18422.620	72			
Corrected total	797.797	71			

^a^ *R* squared = 0.367 (adjusted *R* squared = 0.319).

**Table 3:** Multiple comparison between different perforation sizes.

Tukey HSD^a^^,^ b			
Perforation size	*N*	Subset	
		1	2
1.4	24	13.0738	
0.8	24		16.7359
0	24		17.1275
Sig.		1.000	0.876

*Note:* Means for groups in homogeneous subsets are displayed. Based on observed means. The error term is mean square (error) = 7.653.

^a^ Uses harmonic mean sample size = 24.000.

^b^ Alpha = 0.05.

Comparison of the means of the six groups showed that the push‐out bond strength of the SE1.4 group was not statistically different from the SA1.4 group, but it was lower than the other groups (*p* < 0.05). No statistically significant differences were found among the SA0, SA0.8, SA1.4, SE0, and SE0.8 groups (Tables 1 and 4).

**Table 4:** Mean push‐out bond strength of the six study groups.

Tukey HSD^a^^,^ b			
Group	*N*	Subset	
		1	2
SE1.4	12	11.6384	
SA1.4	12	14.5092	14.5092
SE0.8	12		16.4164
SE0	12		16.9399
SA0.8	12		17.0554
SA0	12		17.3152
Sig.		0.127	0.144

*Note:* Means for groups in homogeneous subsets are displayed. Based on observed means. The error term is mean square (error) = 7.653.

^a^ Uses harmonic mean sample size = 12.000.

^b^ Alpha = 0.05.

**Figure 3:**
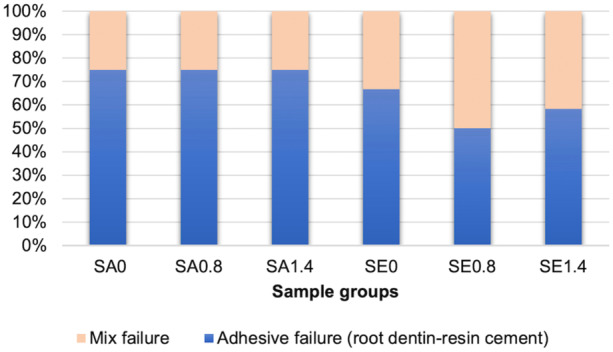
Failure mode of each experimental group.

**Figure 4:**
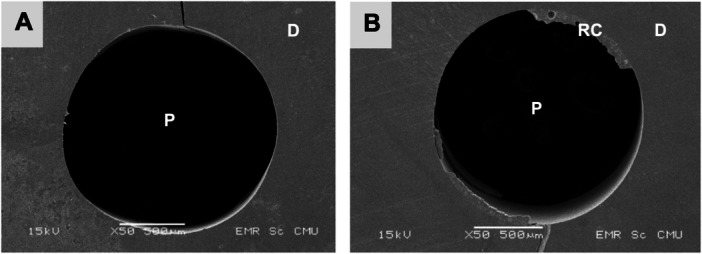
Failure mode exploration using SEM at 50× magnification identified two types of failure: (a) adhesive failure between resin cement and root canal dentin and (b) mixed failure. D, dentin; P, post space; RC, resin cement.

Specimen interface investigation identified two types of failure: adhesive failure between resin cement and root canal dentin, and mixed cohesive and adhesive failure (Figure 3). Examples of both failure types explored using SEM are shown in Figure 4.

## 4 Discussion

This study is the first evaluation of push‐out bond strength in roots that had been repaired by Biodentine. Previous studies only examined MTA, the main component of which is tricalcium silicate (like Biodentine). The results from this study are consistent with the study by Kubo et al. (2013), who reported that there was no significant difference in the bond strength of fiber posts cemented with resin cement and a self‐etch adhesive system between intact roots and roots with 0.9 mm perforations repaired using MTA. The push‐out bond strength of Kubo's study was 2.30–2.81 MPa, which is much lower than this study. This difference may be due to the stronger bond between Biodentine and methacrylate‐based materials compared to that of MTA (Kaup et al. 2015; Cantekin and Avci 2014; Raina et al. 2020). Furthermore, this difference could be caused by different study designs, including the fiber post shape: Kubo et al. used a tapered design that has a lower removal force than a parallel shape of fiber post used in this study (Pruthi et al. 2018). Moreover, the type of resin cement, adhesive used, and UTM settings and/or technique can also affect the results.

The findings of this study indicated that the bond strength observed in the group with a 1.4 mm root perforation was significantly lower than other groups (*p* < 0.05). This result is consistent with the findings reported by Pereira et al. (2016), who found the fiber post bond strength of a 1.5 mm perforation repaired with MTA was 20%–40% lower than the non‐perforated group, depending on the type of resin cement used. Both previous studies and the current study have found that calcium silicate‐based root repair materials, such as MTA and Biodentine, negatively affected bond strength between resin cement and root canal dentin (Pereira et al. 2016; Kubo et al. 2013). The strength of the bond between resin‐based material and dentin (11.07–44.0 MPa) (Wagner et al. 2014; Ebrahimi Chaharom et al. 2017; Lührs et al. 2010; Moghaddas et al. 2017; Teixeira and Chain 2010; Muñoz et al. 2015; Takamizawa et al. 2016) is much higher than resin‐based material and Biodentine (1.2–19.56 MPa) (Odabaş et al. 2013; Altunsoy et al. 2015; Hursh et al. 2019). Therefore, dentin replacement with Biodentine in roots with a 1.4 mm perforation may reduce bond strength compared with a 0.8 mm perforation and no perforation.

Sarkis‐Onofre et al. (2014) published a systematic review on bonding interactions among adhesive systems, resin cements, and root canal dentins. They suggest that FRC post cemented with self‐adhesive resin cement has a good bond strength and is user friendly because there is no need to prepare root canal dentin before cementation. Resin cement used with an etch‐and‐rinse adhesive system has many factors that affect the bond quality to root canal dentin; a crucial factor is infiltration of resin monomer into demineralized dentin, which affects long‐term bond strength (Spencer and Swafford 1999). Therefore, in this study, we used self‐adhesive and self‐etched resin cement, that has the mechanism of demineralization concurrent with resin infiltration, to reduce working steps and technical errors.

In this study, no statistically significant difference (*p* > 0.05) was found in the push‐out bond strength of FRC posts cemented with self‐adhesive resin cement and resin cement coupled with universal adhesive in self‐etched mode in Biodentine‐repaired root canals. This result may be attributed to the fact that both resin cement systems use a phosphoric acid derivative functional monomer to create a chemical bond to root canal dentin, namely MDP in Single Bond Universal Adhesive and methacrylated phosphoric ester in RelyX Unicem (3M, ESPE 2007; 3M, ESPE 2007; Matinlinna 2015). These findings are consistent with those of Ubaldini et al. (2018), who used micro–Raman spectroscopy to evaluate the extent of chemical bonding between dentin and adhesive or resin cement. Their results demonstrated that the degree of chemical interaction correlated more strongly with bond strength than did resin monomer infiltration into the dentinal tubules. Furthermore, they reported that RelyX Unicem 2 and RelyX Ultimate, used with Single Bond Universal adhesive, exhibited comparable levels of chemical bonding.

Analysis of the failure modes revealed two types: adhesive failure at the interface between the resin cement and root canal dentin, and mixed failure. Adhesive failures were mostly found in both resin cement types; this finding implies that the weakest bonding area was the resin cement‐dentin interface in the control groups and the dentin‐Biodentine interface in the perforation groups. Therefore, adhesive failures mean that the push‐out bond strength investigation in this study represents the actual bonding value between resin cement and dentin with or without Biodentine. Mixed failure indicates that the bond strength in some areas between the resin cement and the dentin was stronger than the cohesive strength of the resin cement itself. Adhesive failure between the FRC post and resin cement was not found, perhaps due to proper surface treatment with silane on the FRC post before the bonding procedure, and thus a high bond strength between the resin cement and the FRC post.

There have been good clinical reports for Biodentine, and it is generally used as a root‐repair material (Takamizawa et al. 2016). Of note, this study investigated the influence of Biodentine in a perforated tooth restorative procedure, and the findings suggest that in the case of a large perforation size, the bond between Biodentine and resin cement is low, and thus FRC post debonding may occur. This study tested the push‐out bond strength of FRC posts at 24 h after cementation. The authors suggest that future studies should evaluate specimens after an aging procedure to investigate long‐term bond strength. Moreover, as this investigation was conducted in vitro, its applicability to clinical conditions is inherently limited; therefore, additional studies incorporating conditions that more closely approximate the clinical environment are warranted.

## 5 Conclusions

Based on the limitations of this study, the following conclusions can be drawn.

1. The use of self‐adhesive resin cement and resin cement coupled with universal adhesive in self‐etched mode for luting FRC posts to Biodentine‐repaired root canals did not result in a statistically significant difference in push‐out bond strength.
2. Roots with a 1.4 mm perforation that are repaired with Biodentine showed lower push‐out bond strength of the FRC post, significantly in the SE group and not significantly in the SA group, compared to those with a 0.8 mm perforation and the control group.

## Author Contributions

**Yasudthama Rattana‐aporn:** methodology, investigation, data curation, formal analysis, visualization. **Weeranuch Thong‐ngarm:** conceptualization, supervision, project administration, validation, writing and editing.

## Conflicts of Interest

The authors declare no conflicts of interest.

## Data Availability

The data that support the findings of this study are available from the corresponding author upon reasonable request.
